# Development of the Tiers of Service framework to support system and operational planning for children’s healthcare services

**DOI:** 10.1186/s12913-021-06616-9

**Published:** 2021-07-13

**Authors:** Sina Waibel, Janet Williams, Yasmin Tuff, Joanne Shum, Jennifer Scarr, Maureen O’Donnell

**Affiliations:** 1 Provincial Health Services Authority, Child Health BC, #260 – 1770 West 7th Avenue, BC V6T 1Z3 Vancouver, Canada; 2 Department of Pediatrics, University of British Columbia, Faculty of Medicine, 317 – 2194 Health Sciences Mall, BC V6T 1Z3 Vancouver, Canada

**Keywords:** Health planning, Pediatrics, Quality improvement, Rural health services, Review, Delivery of health care, Quality of health care

## Abstract

**Background:**

Providing access to pediatric healthcare services in British Columbia, Canada, presents unique challenges given low population densities spread across large geographic distances combined with a lack of availability of specialist providers in remote areas, leading to quality of care shortcomings and inequalities in care delivery. The study objective was to develop a framework that provides a common language and methodology for defining and planning child and youth healthcare services across the province.

**Methods:**

The framework was developed in two phases. In Phase 1, a literature and jurisdictional review was completed using the following inclusion criteria: (i) description of a framework focusing on organizing service delivery systems (ii) that supports health service planning, (iii) includes specialty or subspecialty services and (iv) has been published since 2008. In Phase 2, a series of meetings with key provincial stakeholders were held to receive feedback on the developed Tiers of Service framework versions that were based on the literature and jurisdictional review and adjusted to the British Columbian health care context. The final version was endorsed by the Child Health BC Steering Committee.

**Results:**

Ten medical articles and thirteen jurisdictional papers met the established selection criteria and were included in this study. Most frameworks were developed by the Australian national or state jurisdictions and published in jurisdictional papers (n = 8). Frameworks identified in the medical literature were mainly developed in Canada (n = 3) and the US (n = 3) and focused on maternity, neonatal, critical care and oncology services. Based on feedback received from the expert group, the framework was expanded to include community-based services, prevention and health determinants. The final version of the Tiers of Service framework describes the specific services to be delivered at each tier, which are categorized as Tier 1 (community services) through Tier 6 (sub-specialized services). Two consecutive steps were identified to effectively use the framework for operational and system planning: (i) development of a ‘module’ outlining the responsibilities and requirements to be delivered at each tier; and (ii) assessment of services provided at the health care facility against those described in the module, alignment to a specific tier, identification of gaps at the local, regional and provincial level, and implementation of quality improvement initiatives to effectively address the gaps.

**Conclusions:**

The benefits of the Tiers of Service framework and accompanying modules for health service planning are being increasingly recognized. Planning and coordinating pediatric health services across the province will help to optimize flow and improve access to high-quality services for children living in British Columbia.

**Supplementary Information:**

The online version contains supplementary material available at 10.1186/s12913-021-06616-9.

## Background

Providing appropriate access to health care in the Canadian province of British Columbia presents some unique challenges given its low population densities spread across large geographic distances combined with a lack of availability of specialist providers in remote areas [1]. According to the 2016 census, British Columbia has a population of 4.6 million among whom 12 % live in rural areas [2]. The population density is five people per square kilometre [2]. In general, individuals who reside in rural communities tend to have poorer health outcomes and lower socio-economic status compared to their urban counterparts [3].

Twenty % of the total population in British Columbia are children and youth from 0 to 19 years. The highest number of children live in the Lower Mainland (the region surrounding and including Vancouver) but the highest ratio of children to adults is found in the rural and northern parts of British Columbia, where 24 % of the total population are children [4]. Children are particularly vulnerable and investing in the early years of life can improve health and well-being both in midlife and in later years [5]. Although overall health and well-being of children and youth is reasonably good in British Columbia, particularly compared to other jurisdictions in Canada, there are substantial disparities among sex and geography [6].

Five regional or geographic and two provincial health authorities (Provincial Health Services Authority, and First Nations Health Authority) administer hospital or community-based services or both; either by delivering the services directly or by contracting with other health care organizations and providers [7]. Approximately one-third of child medical inpatient care in British Columbia is provided by BC Children’s Hospital, a highly specialized hospital in Vancouver, where the most complex children are usually seen. The remaining two-thirds are offered by hospitals spread across the vast geography of the province.

During the Child Health BC Forum 2008, which brought together representatives from key service sectors and government, the need for developing a unifying framework for child and youth health was identified [8]. This framework would assist in the planning and coordination of services provincially, within and across health authorities and multiple sectors and service providers in British Columbia. It would aim to support provincial collaboration and provide a consistent approach to service planning and delivery across the province [8].

In a recently published quality of care policy framework, the Ministry of Health noted considerable variation in service planning models and use of clinical, staffing, operational and management practices across the health authorities [1]. One of the key actions identified by the Ministry of Health was to consider the role and scope of hospitals in the regional healthcare continuum as well as to clarify the distribution of hospital services [9]. They further recognized the need to outline referral pathways for patients to access higher levels of care in larger population centres across the province [1]. This underscores the importance of creating a framework that could be applied to the whole province and address the quality of care shortcomings identified by the Ministry of Health.

The objective was to develop a framework that provides a common language and methodology for defining and planning child and youth health services in British Columbia.

## Methods

The framework was developed in two phases: (i) review of the literature and jurisdictional papers on organizing service delivery systems, and (ii) organization of a series of interviews with key provincial stakeholders to discuss its applicability to the British Columbian health care system and emerging themes.

### Phase 1: Review of the medical literature and jurisdictional papers

An extensive search for frameworks to define, plan and coordinate healthcare services was initially conducted in 2011. This initial search was updated in 2020 for the purpose of this article to provide a comprehensive and revised list of publications relevant to the topic. The search comprised: (i) websites and relevant documents focusing on health care delivery systems including websites of provincial and national governments, professional governing bodies such as the American College of Surgeons, as well as supra-national bodies such as the World Health Organization. Countries such as Canada, Australia, New Zealand, and the United States were specifically targeted in the search to provide perspectives and experiences from different geographic regions of other developed countries; and (ii) published medical literature (MEDLINE). A comprehensive search strategy was utilized that consisted of a combination of descriptors and keywords related to the research area. Search words included but were not limited to levels of care, tiering, quality improvement frameworks, role delineation or hospital categorization. Titles, abstracts or retrieved full-text articles were scanned for relevance to the research topic. Bibliographies of retrieved full-text articles were hand searched for further relevant references. An additional file describes the search strategy applied to the jurisdictional and medical literature review (see Additional file 1).

Jurisdictional papers and retrieved full-text articles were scanned for adherence to the following inclusion criteria: (i) description of a framework focused on organizing service delivery systems (ii) that supports health service planning, (ii) includes specialty or subspecialty services (for example, pediatric, intensive care, mental, maternity), and (iv) has been published since 2008. We included frameworks of any type of specialty services (for example, maternity services or neonatal services). Excluded from the study were frameworks that: (i) focused on primary care only, (ii) classified facilities or hospitals as a whole as opposed to defining the specific healthcare services that they provide, and (iii) were published in any language other than English. Some of the national and international jurisdictions were contacted to retrieve more specific information about their published framework.

### Phase 2: Interviews with key provincial stakeholder to develop the Tiers of Service framework

An interdisciplinary expert group was established to support the development of a framework that should facilitate the planning of services appropriate to meet the pediatric population’s needs in the province. The participants were selected purposively based on their expertise and experience in pediatric medicine, nursing, allied health and health systems planning. Individual or group phone or in-person interviews were held with the different participants to seek their feedback on various draft versions of the framework, which we developed using the results of the literature review and local knowledge of the British Columbian health care system. There was no formal interview guide or set of questions; participants were asked to provide general feedback on the draft versions. Several rounds of interviews and revisions were necessary until a refined framework was developed that was brought forward to the Child Health BC Steering Committee. This committee consists of representatives from each health authority (geographic and provincial) as well as child-serving ministries and academic partners. Subsequent rounds of revisions were necessary to adjust the framework according to the committee members’ suggestions before we obtained final approval.

### Patient and public involvement

There was no patients or public involvement in the study design or development of the framework. However, patient and family representatives form part of the advisory committees who support interpretation and distribution of results stemming from self-assessments based on the Tiers of Service framework.

## Results

### Results of review of the medical literature and jurisdictional papers

**Fig. 1:**
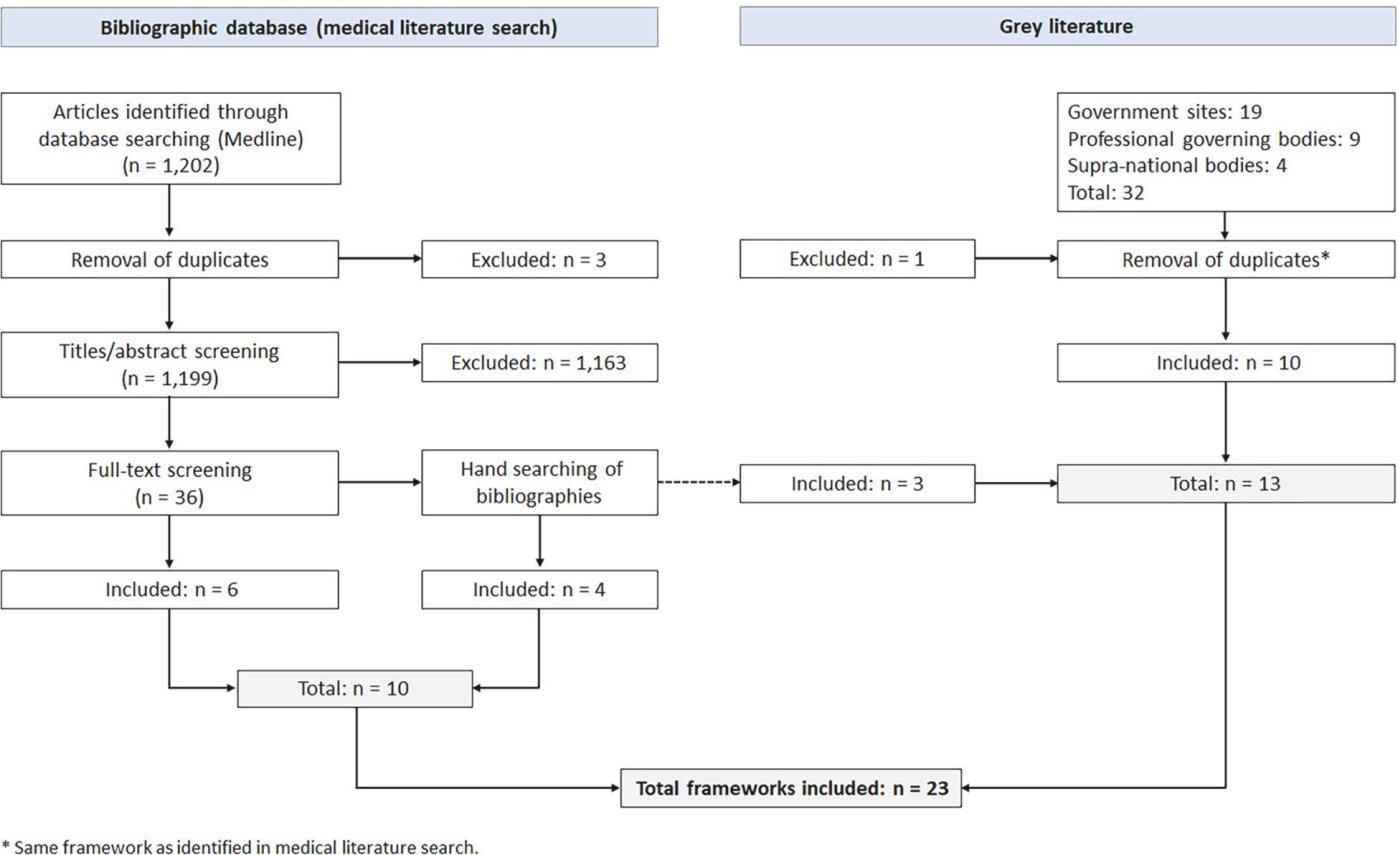
Search results

Ten medical articles and thirteen jurisdictional papers met the established selection criteria and were included in this study (Table 1; Fig. 1). Most of the health service delivery frameworks were developed by the Australian national or state jurisdictions (n = 8), followed by Canada (n = 6) and the US (n = 5). Australia was the most advanced with respect to developing frameworks for organizing service delivery systems with all of its states applying them for service and system planning. The Australian frameworks, often named role delineation or capability framework, were published in jurisdictional papers only (except for one identified also in the Medline search [19]) and comprised services across the continuum of care and for both adults and children. They can be applied to different clinical services, for example anaesthesiology, oncology, geriatrics, medical imaging, or rehabilitation. The remaining frameworks particularly focused on maternity and neonatal services (n = 6), and pediatric (critical care, surgical) services (n = 4). Two frameworks categorized oncology services [18, 22], one framework defined levels of trauma centres [25] and another mental health and addiction services [23].

**Table 1:** Results of review of medical literature and jurisdictional papers on frameworks for organizing service delivery systems

Author(s), Year	Title	Country	Service focus	Proposed framework	Aim	Description
***1. Medical literature***						
American Academy of Pediatrics Committee on Fetus And Newborn [10], 2012	Levels of neonatal care	USA	Neonatal services	Levels of neonatal care	To review the current status of the designation of levels of newborn care definitions in the United States, which were delineated in a 2004 policy statement by the American Academy of Pediatrics [11].	• Updates the previous classification to: basic care (Level I), specialty care (Level II), and subspecialty intensive care (Level III, Level IV). • Each level reflects the minimal capabilities, functional criteria, and provider type required.
Falster et al. [12], 2012	Development of a maternity hospital classification for use in perinatal research	Australia	Maternity services	Maternity hospital classification	To develop a maternity hospital classification, using stable and easily available criteria that would have wide application in maternity services research and allow comparison across state, national and international jurisdictions.	• Uses four dimensions of service level for categorization: neonatal care capability, geography, annual average number of births, and hospital status (public or private). • Classifies maternity hospitals by 13 obstetric groups, which were then collapsed into a set of six groups for analysis (tertiary hospital, hospital with continuous positive airways pressure, all other urban hospitals, large regional hospitals (delivery volume ≥ 1000), all other regional hospitals (< 1000), and private hospitals).
Frankel et al. [13], 2019	Criteria for critical care of infants and children: PICU admission, discharge, and triage practice statement and levels of care guidance	USA	Pediatric services	Levels of care for PICU	To update the American Academy of Pediatrics and Society of Critical Care Medicine’s 2004 Guidelines and levels of care for PICU.	• Distinguishes between three levels of care: quaternary or specialized PICU care (provides regional care and serves large population or has a large catchment area), tertiary PICU care (provides advanced care for many medical and surgical illnesses in infants and children), and community PICU care (provides a broad range of services and resources that may differ based on institution, hospital size, and referral base). • Addresses important specifications for each PICU level of care, including team structure and resources, technology and equipment, education and training, quality metrics, admission and discharge criteria, and indications for transfer to a higher level of care.
Grzybowski et al. [14], 2009	Planning the optimal level of local maternity service for small rural communities: a systems study in British Columbia	Canada	Maternity services	Population isolation model	To develop and apply a population isolation model to define the appropriate level of maternity service for rural communities in British Columbia, Canada.	• Models rural healthcare service based on community population size, social vulnerability, and degree of isolation or access to maternity services (determining the rural birth index score). • Applies the rural birth index score to six community maternity service levels: no local intrapartum service, local intrapartum service without operative delivery, local GP surgical service, mixed model of specialists and GP surgeons, specialist only models.
Kilpatrick et al. [15], 2019	Obstetric care consensus #9: levels of maternal care	USA	Maternity services	Levels of maternal care	To reaffirm the need for levels of maternal care, as initially presented in the 2015 Obstetric Care Consensus [16]. To reaffirm that the goal of levels of maternal care is to reduce maternal morbidity and mortality. To clarify definitions and revise criteria by applying experience from jurisdictions that are actively implementing levels of maternal care.	• Establishes a classification system with levels of maternal care that pertain to: basic care (Level I), specialty care (Level II), subspecialty care (Level III), and regional perinatal health care centers (Level IV), to standardize a complete and integrated system of perinatal regionalization and risk-appropriate maternal care. • Provides definitions, capabilities, and health care providers for each of the four levels of maternal care and for birth centers.
Marshall et al. [17], 2016	What is an intensive care unit? A report of the task force of the World Federation of Societies of Intensive and Critical Care Medicine	Canada and others	Critical care services	-	To develop a globally applicable answer to the question “What is an ICU?”	• Describes three ICU levels: Level 1 ICU is capable of providing oxygen, non-invasive monitoring, and more intensive nursing care than on a ward; Level 2 ICU can provide invasive monitoring and basic life support for a short period; Level 3 ICU provides a full spectrum of monitoring and life support technologies, serves as a regional resource for the care of critically ill patients, and may play an active role in developing the specialty of intensive care through research and education.
Matthey et al. [18], 2010	Facilities for the treatment of adults with haematological malignancies – ‘Levels of Care’: BCSH Haemato-Oncology Task Force 2009	United Kingdom	Oncology services	Levels of care (BCSH Haemato-Oncology Task Force)	To provide an updated guideline for use both by providers of this clinical care and by those who commission it.	• Propose three levels of care with Level 2 being subdivided into Levels 2a and 2b. • Levels of care reflect the facilities and resources required to treat patients with haematological malignancies.
Queensland Government [19], 2016	The clinical services capability framework	Australia	Different clinical services	Clinical services capability framework	References the jurisdictional paper [20], which objective is: to guide a coordinated and integrated approach to health service planning and delivery in Queensland.	• Categorizes clinical services into six levels. Each level builds on the previous level, with Level 1 managing the least complex patients to Level 6 managing the highest level of patient complexity. • Informs health service planning and delivery by providing a set of minimum patient safety criteria by each clinical service area’s capability level.
Saigal et al. [21], 2017	Mapping the characteristics of critical care facilities: assessment, distribution, and level of critical care facilities from Central India	India	Critical care services	-	To classify Intensive Care Units (ICUs) according to the level of care in the state of Madhya Pradesh.	• Provides a consensus scoring grid to define three levels of care of ICUs. • Assigns ten parameters to the scoring grid including number of ICU beds, nurse to patient ratio, invasive ventilator to bed ratio, doctor’s qualification, monitoring devices, imaging, laboratory, procedures conducted, protocols, teaching and training.
Vandenberg et al. [22], 2009	A framework for the organization and delivery of systemic treatment	Canada	Oncology services	Regional model of care for systemic treatment	To provide safe, evidence-based systemic cancer treatment while maximizing the efficient use of resources and implementing the principle of patient-centred care provided as close to home as possible.	• Comprises four levels of care determined by a regional systemic treatment program and three integrated structures (integrated cancer programs, affiliate institutions, and satellite institutions). • Level of complexity and availability of service differentiate one level from another.
***2. Jurisdictional papers***						
American Association of Community Psychiatrists [23], 2009	Level of care utilization system for psychiatric and addiction services (LOCUS): adult version 2010	USA	Mental health and addiction services	Level of care utilization system for psychiatric and addiction services	To describe a continuum of service arrays which vary according to the amount and scope of resources available at each “level” of care.^a^	• Defines six levels of care in the service continuum: recovery maintenance and health management, low intensity community based services, high intensity community based services, medically monitored non-residential services, medically monitored residential services, and medically managed residential services. • Each level is described in terms of four variables: care environment, clinical services, support services and crisis resolution and prevention services.
American College of Surgeons [24], 2015	Optimal resources for children’s surgical care v.1.	USA	Pediatric surgical services	Children’s surgical center levels	To define optimal resources for children’s surgical care.	• Classifies three tiers of children’s surgical centers: basic, advanced, comprehensive. • Identifies criteria that are judged essential for each level of children’s surgical center designation. • Considers medical and access needs of the pediatric population.
American College of Surgeons [25], 2014	Resources for optimal care of the injured patient	USA	Trauma services	Levels of trauma centers	To set appropriate standards for the optimal care of the trauma patient, ensure the right infrastructure and people, ensure high quality data for performance improvement and verify that quality outcomes are present.^a^	• Defines four levels of trauma care facilities: Level I (regional resource trauma center that is a tertiary care facility), Level II (expected to provide initial definitive trauma care, regardless of the severity of injury), Level III (serves communities that do not have immediate access to a Level I or II institution), Level IV (provides advanced trauma life support before patient transfer in remote areas where no higher level of care is available).
Australian Government [26], 2012	National maternity services capability framework	Australia	Maternity services	National maternity services capability framework	To support the provision of safe maternity services in as many localities as possible across Australia in both the public and private sectors.	• Defines six levels of maternity care by describing the minimum service capability requirements for both public and private maternity services across all rural, regional and metropolitan settings. • Identifies four key elements to meet the objectives of service safety, quality, planning and coordination: complexity of care, workforce, clinical support services, and service networks and integration.
Government of South Australia [27], 2016	Clinical services capability framework: fundamentals of the framework	Australia	Different clinical services	Clinical services capability framework	To guide a coordinated and integrated approach to health service planning and delivery in South Australia.^a^	• Categorizes clinical services into six service levels with Level 1 managing the least complex patients and Level 6 managing the highest level of patient complexity. Outlines indicative service requirements, workforce requirements and support services for health services to deliver safe and appropriately supported clinical service delivery.
Government of Western Australia [28], 2015	WA health clinical services framework 2014–2020	Australia	Different clinical services	WA health clinical services framework	To describe medium and long-term horizons and strategic parameters that can be used by individual health services, hospitals, and non-hospital service providers to inform and guide their individual clinical service/s plans.	• Provides a blueprint for the whole health system in planning services, workforce, infrastructure, technology and budgeting. • Gives an overview of the minimum requirements and capabilities necessary to deliver safe and quality services at a particular level in the range one through six (form least to most complex).
National Health Service [29], 2016	Paediatric critical care standards for London: level 1 and 2	United Kingdom	Pediatric critical care services	Paediatric critical care levels	For NHS Trusts: to determine whether current paediatric critical care services meet appropriate standards. For commissioners: to guide their decisions about service provision and quality assurance.	• Distinguishes between three pediatric critical care units: Level 1 (basic critical care), Level 2 (intermediate critical care in a district general hospital or tertiary hospital setting), and Level 3 (advanced critical care). • Provides the pediatric critical care Level 1 and 2 standards.
New Zealand Ministry of Health and District Health Boards [30], 2014	The NZ role delineation model: overview and instructions for use	New Zealand	Different clinical services	New Zealand role delineation model	To differentiate complexity between services within, and across District Health Boards providers.	• Creates specialty service capability levels that can be used to describe and understand patient services across the region. • The six levels of complexity usually extends from community based services through to the most complex setting.
NSW Government [31], 2018	NSW health guide to the role delineation of clinical services	Australia	Different clinical services	Role delineation	To provide a consistent language across NSW for describing clinical services.	• Describes the minimum support services, workforce and other requirements for clinical services to be delivered safely. • Clinical services are categorised into six service levels with Level 1 managing the least complex patients and Level 6 managing the highest level of patient complexity.
Provincial Council for Maternal and Child Health [32], 2018	Paediatric levels of care	Canada	Pediatric services	Paediatric levels of care	To ensure that quality services are provided to paediatric patients and their families as close to home as possible, in other words, care provided in the right place at the right time by the right providers.	• Describes three components: (i) three levels of inpatient care; (ii) classification of paediatric medical and surgical patients by acuity/complexity and procedural complexity; (iii) paediatric requirements related to physician/nursing skills and education needs; organizational/continuous quality improvement requirements; allied health human resource requirements; and diagnostic imaging, procedures, treatments, and equipment.
Provincial Council for Maternal and Child Health [33], 2013	Standardized maternal and newborn levels of care definitions	Canada	Maternity and neonatal services	Levels of maternal-newborn care	None stated	• Describes maternal and newborn services for Levels Ia and Ib (small hospital), Levels IIa, IIb and IIc (large community hospital), and Level III (tertiary hospital). • Highlights that maternal and newborn levels should be aligned (i.e. the levels are the same within each organization).
Tasmanian Government [34], 2018	Tasmanian role delineation framework and clinical services profile	Australia	Different clinical services	Tasmanian role delineation framework	To describe the minimum support services, safety standards, skills and competencies, networking arrangements, and other service requirements necessary to provide a service at a specific level to ensure safe and appropriately supported clinical service delivery.	• Categorizes core clinical services (e.g. medical imaging, or anaesthetics) and clinical support services into six levels of service provision. • The service levels are cumulative and build on each previous level’s capability requirement.
Victorian Government [35], 2017	Statewide design, service and infrastructure plan for Victoria’s health system: 2017–2037	Australia	Different clinical services	System role delineation framework	None stated	• Different capability frameworks, such as the capability framework for Victorian maternity and newborn services [36], specify the minimum requirements each health service must meet to provide safe, high-quality services to patients of varying complexity.

^a^ This is one of several given objectives in the original document

 The number of service levels or tiers used in the frameworks varied between three, four or six. All frameworks elaborated in Australia consisted of six levels, where level 1 manages the least complex patients and level 6 the highest level of complexity. Most of the remaining frameworks focusing on pediatric and critical care services used three levels. In contrast, maternal and neonatal levels of care as well as trauma categorization applied four levels, however, with descriptions differing substantially.

### The Tiers of Service framework

The initial draft of the Tiers of Service framework was developed by the research team based on the identified frameworks in the medical literature but particularly the work published from other jurisdictions, mainly from New South Wales [31] and Queensland [20]. Both, the Role Delineation Framework [31] and Clinical Services Capability Framework [20] describe a set of minimum patient safety criteria for each tier and aim to provide a consistent language across the jurisdiction to use when describing and planning health services.

Our initial framework consisted of four tiers that provide local, community, regional or provincial services, similar to other identified models. Based on the feedback received from the expert group, three aspects were modified: first, service areas were expanded to include community- and hospital-based services; second, prevention was included as a focus in the framework; and third, health determinants were being acknowledged in the framework as being part of a broader context of children’s health.

Apart from the adjustments made to the framework, three important emerging themes arose from the discussions with the expert group and helped to refine and further the understanding of the framework: (i) each tier has a unique role in delivering services within the health care system, (ii) the importance of clearly delineating responsibilities (also called capabilities in some jurisdictions) and requirements (also called resources) – while requirements are much easier to describe both are equally important and interrelated, and (iii) the need to understand the relationship between tiers and the importance of working together as a network across the system.

In September 2016, the Child Health BC Steering Committee endorsed the Tiers of Service framework and agreed to utilize it to improve child and youth health in the province. One last major adjustment was made in February 2017 when the committee decided to use six instead of four tiers. The final version of the Tiers of Service framework describes the responsibilities and requirements for specific pediatric health services to be delivered at each tier (not the hospital or health facility as a whole). The framework should facilitate the planning and development of services appropriate to meet the needs of the relevant catchment area (local, regional and/or provincial). It provides a guide to the responsibilities, requirements and critical mass to provide safe, sustainable and appropriate level of pediatric services across the province. Even though cost-efficiency or control is not one of the main goals, the framework might promote an efficient use of resources by better coordinating services across health authorities.

**Fig. 2:**
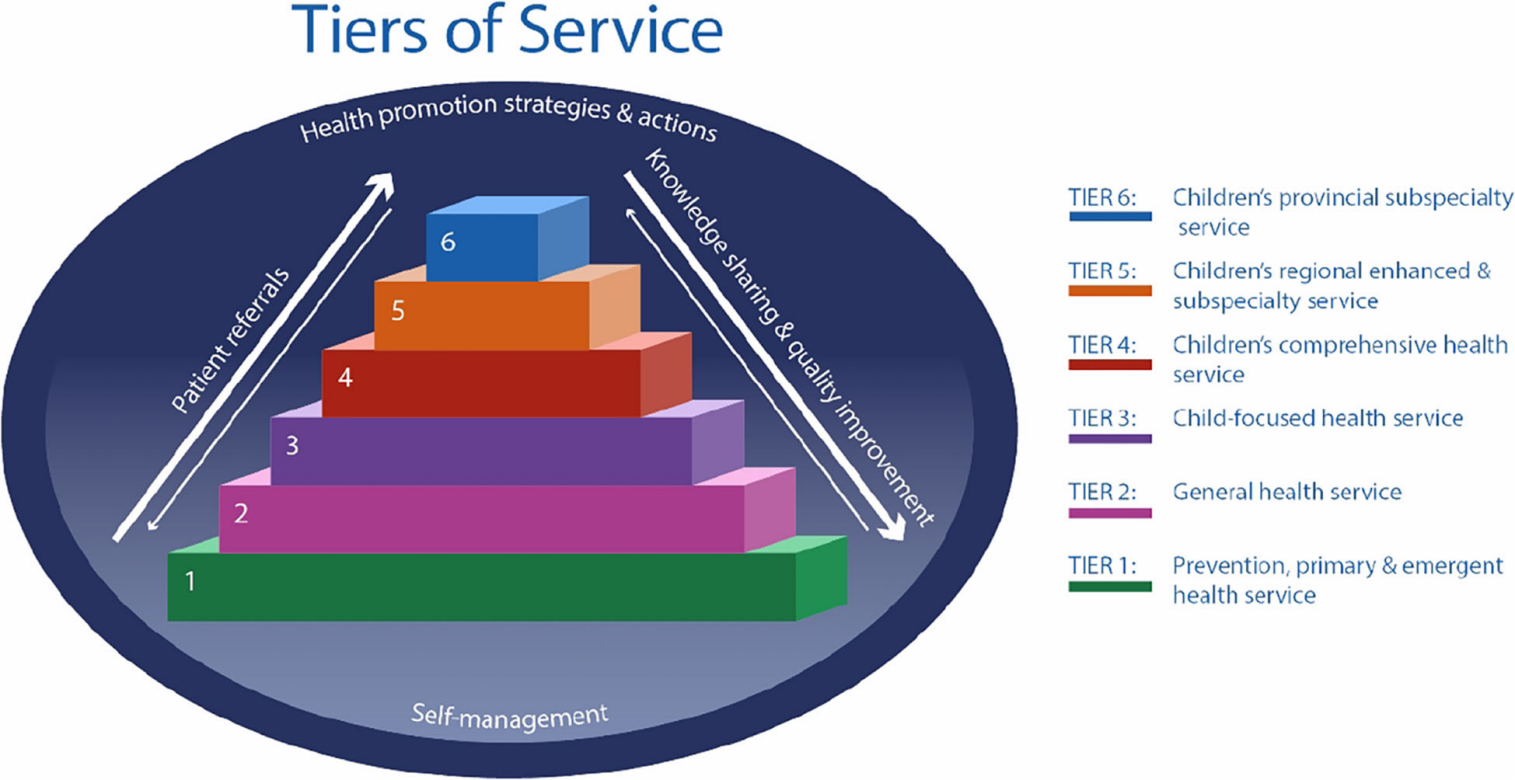
Children’s Tiers of Service framework

In the Tiers of Service framework, the services are categorized as Tiers 1 through 6, with Tier 1 offering a wide breadth of service that is accessible in most communities, targeting health promotion and common, low complexity health needs across the life span (Fig. 2). In comparison, Tier 6 offers in-depth, sub-specialized pediatric-focused services targeting low incidence, high complexity health needs which often require the availability of other on-site subspecialty teams. Each one of these tiers is important and has a unique role in serving children and youth; thus the health system functions best when all tiers accomplish their roles and serve the needs of their population. The framework further recognizes that health services, while important, are one of several factors that contribute to overall child and youth health and wellbeing, next to health promotion strategies and actions as well as patient self-management. An additional file describes each tier in the Children’s Tiers of Service framework in more detail (see Additional file 2).

**Fig. 3:**
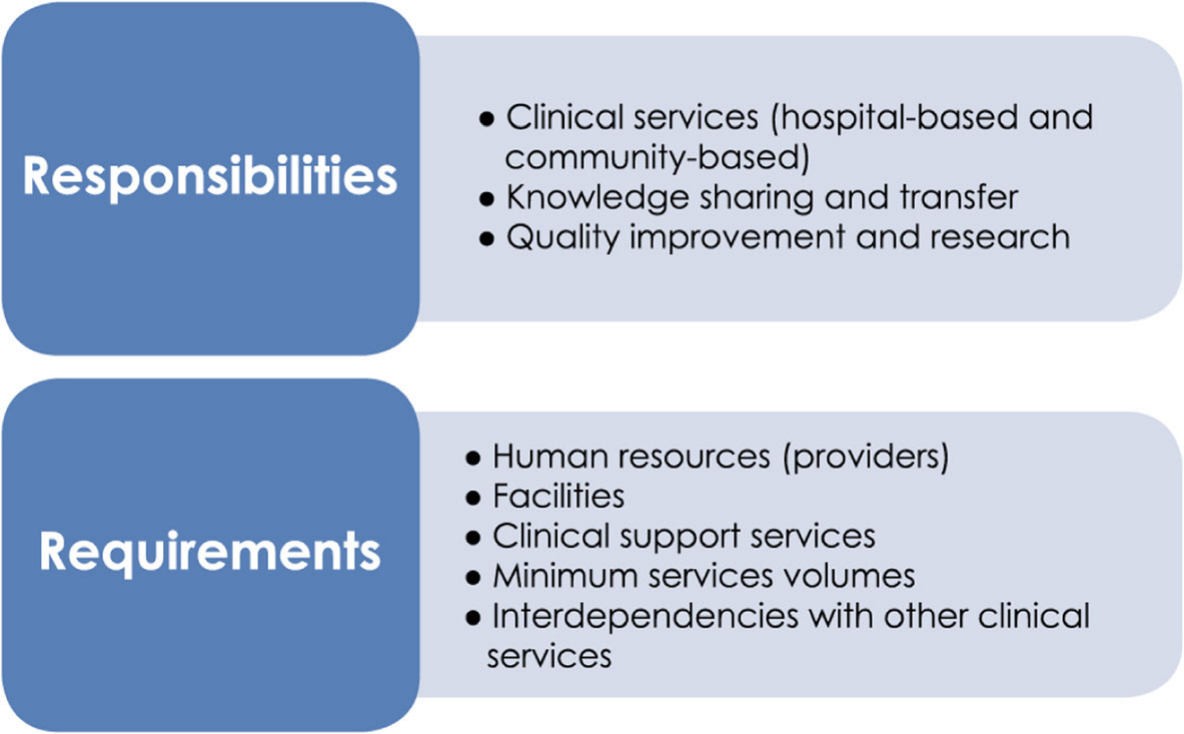
Service provider responsibilities and requirements

In the framework, service descriptors identify different types of provider responsibilities  and requirements; the latter needs to be in place to meet the provider responsibilities (Fig. 3).

### Implementation of the Tiers of Service framework for operational and system planning

Two consecutive steps were identified to effectively use the Tiers of Service framework for operational and system planning.

In the first step, *modules* are created for different types of health services such as children’s medicine, surgery, emergency care, critical care or mental health services. These modules outline the *criteria*, which are the requirements and responsibilities for specific health services that should be delivered at each tier. The number of criteria is distinct for each of the six tiers, with the higher tiers being expected to meet more criteria given that they offer (sub)specialty services. The development of a module is led by a provincial interdisciplinary working group using the best available evidence including reference documents from health professional colleges and provincial health utilization and outcome data where available. The final version of the module is accepted by key partners in the province. An additional file summarizes the different modules that have been completed or are under development (Additional file 3).

In the second step, utilizing the criteria identified in the module, a self-assessment is completed by medical and operational leaders who are able to provide the relevant information about their hospital or health care centre. The self-assessment results are analyzed to align the facility service to a specific tier. In addition, strengths (met criteria) and opportunities for improvement (unmet criteria) are identified at the local, regional and provincial level. Site-specific, health authority and provincial reports are generated to support operational and system planning. Based on the results, quality improvement initiatives are identified, prioritized, implemented and finally evaluated.

## Discussion

The Tiers of Service framework provides a guide to the responsibilities and requirements needed to deliver safe, sustainable and appropriate services at each tier. The framework will facilitate the coordination and integration of health service planning and delivery [20]. A strengthened use of tiered pediatric networks should lead to better health outcomes for patients by enabling right care, in the right place, at the right time [37]. The development of the framework was informed by a review of medical and grey literature from other jurisdictions around the world as well as expert opinions.

Aligning or delineating services to a specific level or tier has become popular in various jurisdictions, particularly in Australia (New South Wales [31], Queensland [19, 20], South Australia [27], Tasmania [34], Victoria [35], and Western Australia [28]). The Tiers of Service framework, informed by the Australian jurisdictions, consists of six tiers, where the higher the tier the more complex services are provided to the population. Furthermore, the service reach increases with the tier: lower tiers serve the local population and higher tiers the health authority or province. Each tier describes the minimum responsibilities and requirements that the facility should be able to provide; with regards to the delivery of clinical services, knowledge sharing and transfer, and quality improvement and research. Other frameworks use different or additional parameters to help classify and distinguish the service into tiers such as geographical location [12], private versus public status [12], or social vulnerability of the health areas [14].

Using a tiered approach for maternal and neonatal services was of particular interest in the identified literature [10, 12, 14, 15, 26, 33]. Our framework presented herein focuses on pediatric services; however, the main features of the framework could also be adapted to characterize adult services. Similarly, Australian jurisdictions [27, 28, 31, 34, 35] and New Zealand [30] successfully proved that their model can be used for a variety of services such as pathology services, geriatric medicine or older person mental health. The Clinical Services Capability Framework, for instance, comprises thirty clinical service modules [20].

The results of the self-assessments have primarily been used for operational planning, i.e. to identify gaps and develop operational quality improvement initiatives at the local, regional and provincial level to address these. For example, a pediatric early warning system to detect children and youth at risk for deterioration has been implemented in emergency departments across the province [38], or pediatric acute intoxication and substance withdrawal guidelines for emergency care settings have been developed.

The Tiers of Service framework has further been applied to support system planning for healthcare services. Health system planning comprises a range of activities that share the goal of improving the efficiency of health service provision or health outcomes, or both [39]. It is usually initiated by the government or service providers (such as public health agencies or hospitals) [39], and is particularly important in a changing environment including changing population characteristics (e.g. growth, cultural diversity or socioeconomic status), emerging clinical evidence and technologies, or higher constraints on health care spending [39, 40]. The outcome of health system planning should be an actionable link between health care needs and resources [39]. Scientific work carried out to support service planning in a specific region is scarce. Similar to the available frameworks, the published studies commonly focus on mapping and classifying maternity care in Australia [41–44]. Another study by Saigal et al. [21] classifies Intensive Care Units in a state of India to improve health system planning and strengthen referral networks.

The Tiers of Service framework has supported system planning in British Columbia in a variety of ways. The results of the tier alignment of the participating facilities support the development of a network of services that can function at its optimum. Specific examples of system planning related to establishing a plan for provincial mental health education and training across the different tiers and developing referral algorithms for children and youth. Future studies are needed to prove the effectiveness of the framework to support system planning. This would contribute to the lack of sound empirical evidence reflecting on recent approaches to healthcare planning at system level [45].

### Limitations

Four limitations should be noted. First, the identified literature that met the inclusion criteria for this study was limited. This topic has not commonly been investigated by the scientific community but is of high interest to health care policy makers and managers. Therefore, we scanned websites of governments to find additional relevant frameworks on organizing service delivery systems. Nevertheless, we might have missed frameworks that used a different language not considered in our search strategy.

Second, the different steps used to implement the Tiers of Service framework are time-consuming when a rigorous and systematic approach is employed. To create a *module* – that includes the different responsibilities and requirements about a specific service for each tier and is based on a broad stakeholder consultation – approximately six months to one year needs to be calculated, while the self-assessment and data analysis add another three months to the process. However, reflecting on and thoroughly evaluating the process, as it is currently underway, as well as adjusting the process based on the evaluation results is intended to make its application more efficient in the future.

Third, a high completion rate of the self-assessment by the selected facilities is necessary to identify regional and provincial gaps. Obtaining their commitment and engagement can be challenging. We achieved a 100 % completion in previous self-assessments, mostly a function of strong, collaborative relationships between Child Health BC and the health authorities. Furthermore, we selected services that were considered priorities by the current Ministry of Health, for example mental health.

Finally, the self-assessment is based on the medical and operational leaders’ best knowledge. No objective measures corroborate the information they provide at the time of the self-assessment. To ensure data accuracy, the self-assessment data is validated first, by experts who form part of the research team and second, by other hospital staff identified by the medical and operational leaders.

## Conclusions

The Tiers of Service framework supports health care providers and managers in their operational and system planning by assessing the services provided in the province and categorizing them into six tiers with varying levels of complexity. Literature published on frameworks and its use is scarce given that health system and service planning is commonly undertaken by governments or service providers (such as public health agencies or hospitals) [39]. Making this information more easily accessible to researchers would potentially accelerate progress in service planning and thus support the delivery of coordinated and high-quality services.

## Supplementary Information

**Additional file 1.** Search strategy. This file describes the applied search strategy for the jurisdictional review (websites) and medical literature review (Medline), to identify published frameworks focusing on organizing service delivery systems.

**Additional file 2.** Description of each tier in the Children’s Tiers of Service framework. This file describes each of the six tiers used in the Children’s Tiers of Service framework in more detail.

**Additional file 3.** Tiers at a Glance. This file provides a summary and alignment of the different child health modules that have been completed or are under development.

## Data Availability

The results of the literature review are presented in Table 1. No datasets were generated or analysed during the current study.
