# Supplementary material for: Development of the Tiers of Service framework to support system and operational planning for children’s healthcare services
Source: BMC Health Serv Res. 2021 Jul 13;21:693. doi: 10.1186/s12913-021-06616-9 (PMC8276838; doi:10.1186/s12913-021-06616-9)
Supplement: Supplementary file 1 — Additional file 1. Search strategy. This file describes the applied search strategy for the jurisdictional review (websites) and medical literature review (Medline), to identify published frameworks focusing on organizing service delivery systems. [file 12913_2021_6616_MOESM1_ESM.docx]

**Additional file 1.** Search strategy

**1. Review of medical literature (Medline)**

("service delivery framework"[All Fields] OR "service delivery levels"[All Fields] OR "levels of service"[All Fields] OR "levels of care"[All Fields] OR "tiering"[All Fields] OR "role delineation"[All Fields] OR "capability framework"[All Fields] OR "hospital typology"[All Fields] OR "hospital categorization"[All Fields] OR "quality improvement frameworks"[All Fields] OR "hospitals, public/classification"[MeSH Terms]) AND "English"[Language] AND 2008/01/01:2019/12/31[Date - Publication]

**2. Review of websites of governments, professional governing bodies and supra-national bodies**

| Country | Institution | Framework | Website |
| --- | --- | --- | --- |
| Australia | Australian Government - Department of Health | National maternity services capability framework | https://www1.health.gov.au/internet/main/publishing.nsf/Content/maternity-pubs-capab |
|  | Government of South Australia | Clinical services capability framework | https://www.sahealth.sa.gov.au/wps/wcm/connect/cee80c804e3d6dc08815d8c09343dd7f/15137+Framework+Modules+WebSec.pdf?MOD=AJPERES&amp;CACHEID=ROOTWORKSPACE-cee80c804e3d6dc08815d8c09343dd7f-niQ10uE |
|  | Government of Western Australia | WA health clinical services framework | https://ww2.health.wa.gov.au/Reports-and-publications/WA-Health-Clinical-Services-Framework-2014-2024 |
|  | NSW Government | Role delineation of clinical services | https://www.health.nsw.gov.au/services/Pages/role-delineation-of-clinical-services.aspx |
|  | Queensland Government | Clinical services capability framework | https://www.health.qld.gov.au/clinical-practice/guidelines-procedures/service-delivery/cscf |
|  | Tasmanian Government | Tasmanian role delineation framework | https://www.dhhs.tas.gov.au/__data/assets/pdf_file/0009/354465/TRDF_CSP_V_4.0_FINAL.pdf |
|  | Victorian Government Department of Health | System role delineation (including the capability frameworks such as the capability framework for Victorian maternity and newborn services) | https://www2.health.vic.gov.au/hospitals-and-health-services/health-system-design-planning/statewide-plan  https://www2.health.vic.gov.au/hospitals-and-health-services/patient-care/perinatal-reproductive/maternity-newborn-services/maternity-newborn-care |
| Canada | Canadian Paediatric Society | None identified | https://www.cps.ca/ |
|  | Provincial Council for Maternal and Child Health | Paediatric levels of care | http://www.pcmch.on.ca/health-care-providers/paediatric-care/pcmch-strategies-and-initiatives/paediatric-levels-of-care/ |
|  | Provincial Council for Maternal and Child Health | Levels of maternal-newborn care | https://www.pcmch.on.ca/health-care-providers/maternity-care/pcmch-reports-and-recommendations/loc/ |
|  | Other provinces of Canada | None identified |  |
| New Zealand | New Zealand Ministry of Health and District Health Boards | New Zealand role delineation model | https://www.moh.govt.nz/NoteBook/nbbooks.nsf/0/43C4C9001223BCBDCC257B10007AC681/$file/trends-service-design-new-models-care-jul2010.pdf |
| United Kingdom | National Health Services (NHS) | Paediatric critical care levels | https://www.healthylondon.org/wp-content/uploads/2017/10/Paediatric-critical-care-standards-Level-1-and-2.pdf |
| USA | American Association for Community Psychiatry | Level of care utilization system for psychiatric and addiction services (LOCUS) | <https://www.communitypsychiatry.org/resources/locus> |
|  | American Academy of Pediatrics | None identified | https://www.aap.org/en-us |
|  | American College of Obstetricians and Gynecologist | Levels of maternal care | <https://www.acog.org/clinical/clinical-guidance/obstetric-care->consensus/articles/2019/08/levels-of-maternal-care |
|  | American College of Pediatricians | None identified | <https://acpeds.org/> |
|  | American College of Surgeons | Children’s surgical center levels | <https://www.facs.org/Quality-Programs/Childrens-Surgery/Childrens-Surgery-Verification/standards> |
|  | American College of Surgeons | Levels of trauma centres | <https://www.facs.org/Quality-Programs/Trauma/TQP/center-programs/VRC/resources> |
|  | Academic Pediatric Association | None identified | <https://www.academicpeds.org/> |
|  | American Pediatric Society | None identified | <https://www.aps1888.org/> |
|  | Federation of Pediatric Organizations | None identified | <https://fopo.org/> |
| Supra-national bodies | World Health Organization, including the European Observatory on Health Systems and Policies | None identified | <https://www.who.int/> |
|  | Pan American Health Organization | None identified | <https://www.paho.org/> |
|  | World Bank | None identified | <https://www.worldbank.org/> |
|  | European Commission | None identified | <https://ec.europa.eu/info/index_en> |
