# Supplementary material for: Development of the Tiers of Service framework to support system and operational planning for children’s healthcare services
Source: BMC Health Serv Res. 2021 Jul 13;21:693. doi: 10.1186/s12913-021-06616-9 (PMC8276838; doi:10.1186/s12913-021-06616-9)
Supplement: Supplementary file 2 — Additional file 2. Description of each tier in the Children’s Tiers of Service framework. This file describes each of the six tiers used in the Children’s Tiers of Service framework in more detail. [file 12913_2021_6616_MOESM2_ESM.pdf]

Additional file 2. Description of each tier in the Children's Tiers of Service framework

Tier	Description
**Tier 1**	**Prevention, primary and emergent health service**<br>• Service is accessible in most communities.<br>• Service providers generally serve adults as well as children and youth.<br>• Service targets health promotion and common, low complexity health needs.<br>• Service participates in regional and provincial child health quality improvement initiatives.
**Tier 2**	**General health service**<br>• Service is accessible to multiple communities and/or a local health area.<br>• Service providers generally serve adults as well as children and youth.<br>• Service targets common, low complexity health needs.<br>• Service participates in regional and provincial child health quality improvement initiatives
**Tier 3**	**Child-focused health service**<br>• Service is accessible to multiple local health areas/health service delivery area.<br>• Service providers focus on children and youth, although some may also serve adults.<br>• Service targets relatively common, medium complexity health needs.<br>• Service participates in regional and provincial child health quality improvement initiatives.
**Tier 4**	**Children's comprehensive health service**<br>• Service is accessible to a health service delivery area/health authority.<br>• Service providers focus the majority of their time on children and youth.<br>• Service targets a broad range of medium complexity health needs.<br>• Service offers general child health experiences/placements for a broad range of undergraduate, graduate and post-graduate health care students and residents. Teaching focus is on general pediatrics and child health care.<br>• Service identifies relevant regional child health quality indicators and leads/participates in regional and provincial child health quality improvement initiatives.
**Tier 5**	**Children's regional enhanced & subspecialty health service**<br>• Service is accessible to a health authority.<br>• Most service providers work exclusively with children and youth.<br>• Service targets relatively common, high complexity health needs. Level of complexity usually does not require the availability of other on-site subspecialty teams.<br>• Service offers general child health experiences/placements for a broad range of undergraduate, graduate and post-graduate health care students and residents. Also offers placements for fellows in selected subspecialties.<br>• Service identifies relevant regional child health quality indicators and leads/participates in regional and provincial child health quality improvement initiatives.<br>• Service participates in relevant child health related research.
**Tier 6**	**Provincial subspecialty children's health service**<br>• Service is accessible to the province.<br>• Service providers work exclusively with children and youth.

Tier	Description
	• Service targets low incidence, high complexity health needs. Level of complexity often requires the availability of other onsite subspecialty teams.<br>• Service offers general and subspecialty child health experiences/placements for a broad range of undergraduate, graduate and post-graduate health care students, residents and subspecialty fellows.<br>• Service identifies relevant provincial child health quality indicators and leads provincial quality improvement initiatives.<br>• Service conducts and supports others to conduct relevant child health-related research.
