# Supplementary material for: Development of the Tiers of Service framework to support system and operational planning for children’s healthcare services
Source: BMC Health Serv Res. 2021 Jul 13;21:693. doi: 10.1186/s12913-021-06616-9 (PMC8276838; doi:10.1186/s12913-021-06616-9)
Supplement: Supplementary file 3 — Additional file 3. Tiers at a Glance. This file provides a summary and alignment of the different child health modules that have been completed or are under development. [file 12913_2021_6616_MOESM3_ESM.pdf]

Children's Health Care Services - Tiers at a Glance

1. Clinical Services

Module		Prevention, Primary & Emergent Health Service<br>T1	General Health Service<br>T2	Child-Focused Health Service<br>T3	Children's Comprehensive Health Service<br>T4	Children's Regional Enhanced & Subspecialty Health Service<br>T5	Children's Provincial Subspecialty Health Service<br>T6
Service reach		Community health service area(s).	Community health service area(s)/local health area.	Multiple local health areas/ health service delivery area.	Health service delivery area/health authority.	Health authority.	Province.
Service focus		Supports the health & well-being of infants, children, youth & their families.<br><br>Local services for emergent care.<br><br>Stabilizes & refers/transfers as necessary.	Diagnoses & provides definitive treatment for children with low acuity/complexity conditions & minor, uncomplicated single system injuries.<br><br>Stabilizes & refers/transfers as necessary.	Diagnoses & provides definitive treatment for children with relatively common, medium acuity/complexity conditions & uncomplicated single system injuries.<br><br>Stabilizes & refers/transfers as necessary.	Diagnoses & provides definitive treatment for children with a broad range of medium acuity/complexity conditions (including complex psychosocial issues) & non-life-threatening single & two-system injuries.<br><br>Stabilizes & refers/transfers as necessary.	Diagnoses & provides definitive treatment for children with high acuity &/or relatively common high complexity conditions (including complex psychosocial issues) & single & two-system injuries. The range of conditions is dependent upon the types of subspecialists available.<br><br>Stabilizes & refers/transfers as necessary.	Diagnoses & provides definitive treatment for children with all types of high acuity/complexity conditions (including complex psychosocial issues) & multiple-system injuries, many of whom require care from multiple subspecialty teams.<br><br>Provincial pediatric trauma centre.
ED		Health Centre<br>(<24 hrs/day).	24/7 ED	24/7 ED	24/7 ED	24/7 ED with dedicated space/focus on children	24/7 pediatric ED
Children's Medical Services<br><br>*(General & Subspecialty Medicine)*	Inpatients		Limited capacity for short-term inpatient stays (in the ED or a general inpatient bed). No <u>dedicated</u> pediatric inpatient beds.<br><br>If child in hospital, FP/NP on-call 24/7.	Dedicated pediatric inpatient beds.<br><br>Pediatrician on-call 24/7.	Dedicated pediatric inpatient unit.<br><br>Pediatrician on-call 24/7.	Dedicated pediatric inpatient unit.<br><br>Pediatrician (or resident) <u>on-site</u> 24/7.<br><br>Pediatric subspecialists are available for on-site consultation in higher volume subspecialties which includes but is not limited to neurology & cardiology. Availability is typically days, M-F.	Dedicated pediatric inpatient units, grouped by specialties/ subspecialties.<br><br>Pediatrician (or resident) <u>on-site</u> 24/7.<br><br>Full range of pediatric subspecialists available for on-site patient management & consultation 24/7.
	Outpatients		Clinic space & infrastructure available for visiting specialists & virtual care consultations (in the ED, hospital outpatient or community-based clinic).	Clearly describable process in place to manage children discharged from hospital or ED requiring short-term follow-up by a pediatrician.<br><br>Child-friendly treatment/ procedure space & infrastructure. May be shared with adults.	Outpatient clinics:<br>• General pediatrics<br>• Child maltreatment (non-acute)<br><br>Child-friendly clinic(s) & outpatient treatment/ procedure space & infrastructure. May be shared with adults.	Same as T4 plus:<br><br>Regularly occurring pediatric subspecialty clinics available on-site for higher volume subspecialties which include but are not limited to:<br>• Cardiology<br>• Diabetes<br>• GI medicine<br>• Neurology	Broad range of pediatric specialty/subspecialty clinics on-site.<br><br>Coordinates & provides pediatric subspecialty outreach clinics (on-site or virtual care) throughout the province.

Children's Health Care Services - Tiers at a Glance

Module		Prevention, Primary & Emergent Health Service	General Health Service	Child-Focused Health Service	Children's Comprehensive Health Service	Children's Regional Enhanced & Subspecialty Health Service	Children's Provincial Subspecialty Health Service
		T1	T2	T3	T4	T5	T6
Children's Medical Services cont'd	Outpatients cont'd					Pediatric subspecialty clinics may be staffed by local pediatric subspecialty providers or via outreach from T6.<br><br>Pediatric outpatient clinic and treatment/procedure space is used exclusively by children.	
	Community -based	Promotes healthy infant, child & youth development, injury prevention & parenting.<br><br>Provides immunizations.<br><br>Screens, supports & refers children for developmental delays or other health issues to appropriate resource(s) for assessment.		Assessment & community-based follow-up of children referred for vulnerabilities, delays & other health issues identified through screening.<br><br>Youth-specific drop-in health care services.	Advanced assessment, intervention & follow-up of referred children *living within the HA/HSDA* with hearing loss.		
Children's Surgical Services<br><br>*(Adult & Pediatric Surgical Specialties)*	Procedures		On-site surgical capacity exists (locally or via outreach) for:<br>• Low complexity procedures on a planned, day care basis on healthy children ages 2 & over (ASA 1-2).<br>• Life & limb procedures.	On-site surgical capacity exists for:<br>• Low complexity procedures on a planned & unplanned, inpatient & day care basis on healthy children ages 2 & over (ASA 1-2)<br>• Life & limb procedures.	On-site surgical capacity exists for:<br>• Low complexity procedures on a planned & unplanned, inpatient & day care basis on healthy children ages 6 months & over[1] (ASA 1 - 2).<br>• Life & limb procedures.	On-site surgical capacity exists for:<br>• Medium & selected high complexity procedures (when relevant pediatric surgery specialist is available) on a planned & unplanned, inpatient & day care basis on children of any age, including those with modest medical complexities (ASA 3).[2]<br>• Life & limb procedures.	On-site surgical capacity exists for:<br>• High complexity procedures on a planned & unplanned, inpatient & day care basis on children of any age, including those with high medical complexities (ASA 4-5).

[1] Assumes availability of appropriately credentialled anesthesiologist(s) as per provincial privileging document. This requires an anesthesiologist that has recent experience providing anesthesia to children in the 6 mos - 2 year age group + 10 CPD credits/year in pediatric anesthesiology.

[2] Assumes availability of appropriately credentialled anesthesiologist(s) as per provincial privileging document. This requires an anesthesiologist who has completed a 12-month fellowship in pediatric anesthesia and has recent experience working with children in the 0 - 6 mos age group + 80 CPD credits/yr with at least 20 CPD credits in pediatric anesthesiology. For children ages 6 mos - 2 yrs, see footnote above.

Children's Health Care Services - Tiers at a Glance

Module		Prevention, Primary & Emergent Health Service<br>T1	General Health Service<br>T2	Child-Focused Health Service<br>T3	Children's Comprehensive Health Service<br>T4	Children's Regional Enhanced & Subspecialty Health Service<br>T5	Children's Provincial Subspecialty Health Service<br>T6
Children's Surgical Services cont'd	Surgical Specialties		*Surgical specialties:*<br>Variable, depending on local surgeon availability.<br><br>General surgeon or family practice physician with enhanced surgical skills available in rural & remote sites (not 24/7).<br><br>*Anesthesia:*<br>Anesthesia provider (specialist or family practice physician) available during times surgical procedures are performed.<br><br>Transfer algorithm in place when surgical or anesthesia provider is not available.	*Surgical specialties:*<br>General surgeon on-call 24/7.<br><br>Strive to have dental surgery, ophthalmology, orthopedics, ENT, plastics and urology on-call 24/7.<br><br>Transfer algorithm in place at times appropriate surgical specialty is not available (e.g., vacations).<br><br>*Anesthesia:*<br>Anesthesia provider (specialist or family practice physician) on-call 24/7.	*Surgical specialties:*<br>Specialists on-call 24/7 & available to assess & manage children with all types of surgical conditions except cardiac or neurosurgery-related. Managing a surgical condition may include performing a surgical procedure, developing an alternative management plan or transferring the child to a T5/T6 service.<br><br>*Anesthesia:*<br>Anesthesiologist who meets the age-specific credentialling requirements available on-call 24/7 to provide anesthesia to children ages 6 mos - 2 yrs.	*Surgical specialties:*<br>Specialists available on-call 24/7 & available to assess & manage children with all types of surgical conditions except cardiac or neurosurgery-related. Managing a surgical condition may include performing a surgical procedure, developing an alternative management plan or transferring the child to a T6 service.<br><br>Pediatric surgical specialists available for some specialties (not 24/7).<br><br>*Anesthesia:*<br>Pediatric anesthesiologist on-call 24/7.<br><br>*Outpatients:*<br>Some specialty-specific outpatient clinics available for children with complex needs.	Surgical specialties:<br>Pediatric surgical specialists on-call 24/7 & available to assess & definitively manage children with all types of surgical conditions, including multi-system trauma.<br><br>*Anesthesia:*<br>Pediatric anesthesiologist(s) available 24/7.<br><br>*Outpatients:*<br>Broad range of specialty-specific outpatient clinics available for children with complex needs.
Children's Critical Care (CC) Services					CC service primarily for adults with a limited capacity to serve children.<br><br>All T4 sites:<br>• All ages: Resuscitation & initial stabilization.<br>• 14 - 16.9 yrs, healthy children with new onset "adult" conditions: Full range of CC services.<br><br>Plus, for children not listed in the groups above:<br>• T4 "close sites" (≤2 hrs by road from T5/T6): Provides a limited range of CC services while awaiting arrival of transport team.<br>• T4 "distant sites" (>2 hrs by road from T5/T6): Provides a limited range of critical care services to children with conditions which are expected to resolve quickly (within 24 - 28 hrs).	Regional CC service for children. Full range of CC services.	Provincial CC service for children. Full range of CC services, including an extended range of monitoring & therapeutic interventions. Multiple pediatric subspecialists often involved.

Children's Health Care Services - Tiers at a Glance

Module		Prevention, Primary & Emergent Health Service	General Health Service	Child-Focused Health Service	Children's Comprehensive Health Service	Children's Regional Enhanced & Subspecialty Health Service	Children's Provincial Subspecialty Health Service
		T1	T2	T3	T4	T5	T6
Child Development, Habilitation & Rehabilitation Services<br><br>Focus on Children with Primary Neuromotor Impairment	Child develop-ment & habilitation			Developmental/ functional assessment, goal setting & care planning, intervention, education, transition support, psychosocial support, service coordination & monitoring provided in an outpatient/community-based setting by one or more members of a transdisciplinary or interdisciplinary team.		Subspecialized developmental/ functional assessment, goal setting & care planning, intervention, education, transition support, psychosocial support, service coordination & monitoring in "designated areas of focus" provided in/from regional outpatient/community-based center(s) by a subspecialty interdisciplinary team.	Subspecialized developmental/functional assessment, goal setting & care planning, intervention, education, transition support, psychosocial support, service coordination & monitoring provided in/from a provincial outpatient/community-based setting center by pediatric subspecialty interdisciplinary team(s). Blocks of high dose (intensity/frequency) inpatient/day patient habilitation interventions also available.
	Rehab-ilitation			In collaboration with T5+/- T6, one or more members of a transdisciplinary or interdisciplinary team provide community-based, timely & time-bound, developmentally-tailored general rehabilitation services & psychosocial support. Reintegrates into habilitation service as required.		Timely & time-bound services in designated areas of focus provided in/from a regional center(s) by a subspecialty interdisciplinary team. Reintegrates into habilitation service as required.	In collaboration with T3 +/- T5, pediatric subspecialty interdisciplinary team provides timely & time-bound developmentally-tailored complex subspecialized rehabilitation services & psychosocial support in/from a provincial centre. Reintegrates & supports T3 rehabilitation or habilitation outpatient services as required.
Children' s Mental Health Services<br><br>DRAFT pending acceptance from MCFD	Hospital Inpatients		Short-term inpatient stays for children/youth up to age 18.9 yrs. Accommodated in a *non-pediatric-specific* bed on a *general* inpatient unit. Service focus is on stabilization & crisis intervention. Anticipated length of stay is <72 hrs. By 72 hrs, child/youth will be discharged home with appropriate community MH services or transferred to higher tier. Applicable to rural/remote hospitals.	Inpatient stays for children/youth up to age 16.9 yrs. Accommodated in a *pediatric-specific bed* on a *general* inpatient unit. Service focus is on stabilization & crisis intervention. Anticipated length of stay is <72 hrs. By 72 hrs, child/youth will be discharged home with appropriate community MH services or transferred to higher tier.	Where no T5 specialized child & adolescent psychiatry unit exists *locally* (i.e., within the same community), inpatient stays for children/youth up to age 16.9 yrs. Accommodated on a *pediatric-specific* inpatient unit. Service focus is on stabilization & crisis intervention. Anticipated length of stay is <72 hrs. By 72 hrs, child/youth will be discharged home with appropriate community MH services or transferred to higher tier.<br><br>Where T5 child & adolescent psychiatry unit exists *locally*, admission is arranged to this specialized unit.	Inpatient stays for youth up to age 18.9 yrs. Accommodated on a specialized *child & adolescent* psychiatry unit.<br><br>Service focus is:<br>• Children up to 11.9 yrs: Stabilization & crisis intervention. Length of stay may be >72 hrs.<br>• Children 12 - 18.9 yrs: Stabilization & crisis intervention & ongoing treatment.	Inpatient stays for children/youth up to age 18.9 yrs. Accommodated on one of several *subspecialty child & adolescent psychiatry inpatient units.*<br><br>Service focus includes stabilization & crisis intervention & ongoing treatment for children/youth of all ages.

Children's Health Care Services - Tiers at a Glance

Module		Prevention, Primary & Emergent Health Service	General Health Service	Child-Focused Health Service	Children's Comprehensive Health Service	Children's Regional Enhanced & Subspecialty Health Service	Children's Provincial Subspecialty Health Service
		T1	T2	T3	T4	T5	T6
Children' s Mental Health Services cont'd	Hospital Inpatients cont'd			Clearly describable process is in place for managing youth ages 17 - 18.9 yrs with MH conditions on a general inpatient or alternative unit.	Clearly describable process is in place for managing youth ages 17 - 18.9 yrs with MH conditions on a general inpatient or alternative unit.		
	Community -based & ambulatory			*Community-based providers* provide psychoeducation, skill building & coaching to support recovery/ coping.<br><br>Support access to follow-up care for MH &/or medical condition(s).<br><br>Services may be provided in a range of settings such as child/youth's home, school or an office in the community.	*Community-based interdisciplinary Child & Youth MH (CYMH) Teams* assess, diagnose & treat children/youth with a *broad range of moderate acuity/complexity* MH conditions/concurrent disorders. Teams provide case management & service coordination for children/youth involved with the service.<br><br>Where sufficient volumes exist (i.e., urban settings), dedicated teams provide short-term, assessment & crises intervention outreach services (e.g., in home or in community settings). Where volumes are *insufficient*, a clearly describable process exists for providing these services in alternative ways (e.g., virtually or in local ED).	*Community or hospital outpatient-based, interdisciplinary teams of subspecialty MH providers* assess, diagnose & treat children/youth with *relatively common high acuity &/or high complexity* MH conditions/concurrent disorders. Most children/youth will return to T4 for ongoing follow-up.<br><br>Teams/clinics include but are not limited to:<br>• Infant psychiatry (5 yrs old & younger)<br>• Eating disorders<br>• Externalizing behavioural disorders<br>• Mood/anxiety<br>• Neurodevelopmental disorders with co-morbid MH condition(s).<br><br>Where sufficient volumes exist (i.e., urban settings), home-based & day treatment services are available.	*Hospital outpatient-based, interdisciplinary, subspecialty MH teams* assess, diagnose & treat children/youth with a *broad range of high acuity &/or high complexity* MH conditions/concurrent disorders. Focus is on children & youth with severe, complex &/or persistent MH conditions which have not responded with T2-T5 services. Medical co-morbidities often present & require monitoring/ treatment by one or more medical/surgical pediatric subspecialists. Most children/youth will return to T4 for ongoing follow-up.
	Residential				*Residential placement* in a foster family, kinship or group home for children and youth in Ministry of Children & Family Development (MCFD) care. Placements are not specific to children/youth with MH conditions +/- behavioural concerns.<br><br>MH assessment & treatment services required while in T4 residential placement are provided through community-based & ambulatory services.	*Residential assessment & treatment* service provided in a specialized, staffed group home. i.e., MCFD-contracted Complex Care Community Residential Resource.<br><br>Service focuses on behaviour stabilization & on teaching children/youth/families about techniques for managing challenging behaviours at home.	*Residential assessment & treatment service* provided in a community-based, facility setting. Includes a unit which provides *step-up* (avoid hospitalization) and *step-down* (transition out of hospital care) care.<br><br>Service focuses on children & youth with complex & often co-occurring & recurring conditions.
Children's Substance Use Services		TBD	TBD	TBD	TBD	TBD	TBD
Children's Home-based Services		TBD	TBD	TBD	TBD	TBD	TBD

Children's Health Care Services - Tiers at a Glance

2. Clinical Diagnostic & Therapeutic Services

Clinical Diagnostic Module	Prevention, Primary & Emergent Health Service	General Health Service	Child-Focused Health Service	Children's Comprehensive Health Service	Children's Regional Enhanced & Subspecialty Health Service	Children's Provincial Subspecialty Health Service
	A	B	C	D	E	F
Children's Laboratory, Pathology & Transfusion Medicine	Maternal/fetal, neonatal & pediatric lab collections.<br><br>Minimal, if any, on-site lab testing other than point of care testing (POCT).	Maternal/fetal, neonatal & pediatric lab collections.<br><br>Routine laboratory testing & resulting on-site.<br><br>Limited blood products & components are stored on-site for emergency use.	Same as Tier B plus:<br><br>Routine lab testing, resulting & interpretation on-site (broader range of tests than at Tier B).<br><br>Standard blood & blood components stored on-site (all RBC groups, plasma, albumin, RhIG).	Same as Tier C plus:<br><br>Routine & some specialty lab testing, resulting, interpretation & consultation on-site.<br><br>Some specialized blood components & blood products stored on-site.<br><br>Pools & aliquots blood components for routine, non-emergency transfusions on days, M-F.	Same as Tier D plus:<br><br>Routine & some specialty lab testing, resulting, interpretation & consultation on-site (broader range of testing than at Tier D).<br><br>Some specialized transfusion medicine services available on-site (broader range than at Tier D), including emergency neonatal exchange transfusions.<br><br>Pools & aliquots blood components. Prepares plasma for routine, non-emergency & emergency transfusions 24/7.	Same as Tier E plus:<br><br>Routine & specialty maternal/fetal, neonatal & pediatric laboratory & pathology testing, resulting, interpretation & consultation on-site.<br><br>Routine & specialty maternal/fetal, neonatal & pediatric transfusion medicine services available on-site including intrauterine transfusions and pediatric exchange transfusions.
Children's Medical Imaging	TBD	TBD	TBD	TBD	TBD	TBD
Children's Pharmacy Services	TBD	TBD	TBD	TBD	TBD	TBD

3. Knowledge Sharing & Transfer/Training

Prevention, Primary & Emergent Health Service	General Health Service	Child-Focused Health Service	Children's Designated Health Services	Children's Enhanced & Regional Subspecialty Health Services	Children's Provincial Subspecialty Health Services
T1	T2	T3	T4	T5	T6
Facilitates access to learning activities that support the maintenance of competencies in child health.	Facilitates access to learning activities that support the maintenance of competencies in child health.	Creates or facilitates access to learning activities that support the maintenance of competencies in child health, including the practice of critical clinical skills (e.g., simulation, clinical experience with T4-T6 service).	If T5 service does not exist within the HA, provides HA leadership for pediatric clinical care, education & system planning.<br><br>Organizes regional activities that support the maintenance of child health competencies. e.g., child health rounds and conferences, clinical experiences on-site or via simulation. If T5 exists within the HA, works in conjunction with T5.<br><br>Provides child health experiences/placements for a broad range of undergraduate, graduate & post-graduate health care students & residents.	Same as T4 plus:<br><br>Provides HA leadership for pediatric clinical care, education & system planning.<br><br>Organizes regional activities that support the maintenance of competencies in child health, in conjunction with T4.<br><br>Provides child health experiences/ placements for fellows in selected, higher volume pediatric subspecialties.	Provides provincial leadership for pediatric clinical care, education, research & system planning.<br><br>Pediatric subspecialists provide telephone consultation to health care providers throughout the province 24/7. RNs, allied health & other specialty/subspecialty team members available for consultation days, M-F.<br><br>Organizes provincial activities that support the maintenance of competencies in child health.

Children's Health Care Services - Tiers at a Glance

3. Knowledge Sharing & Transfer/Training cont'd

Prevention, Primary & Emergent Health Service	General Health Service	Child-Focused Health Service	Children's Designated Health Services	Children's Enhanced & Regional Subspecialty Health Services	Children's Provincial Subspecialty Health Services
T1	T2	T3	T4	T5	T6
					Provides child health clinical experiences for T1-T5 staff & physicians throughout the province (on-site &/or via simulation).<br><br>Provides child health experiences/placements for a broad range of undergraduate, graduate & post-graduate health care students, residents & subspecialty fellows.

4. Quality Improvement & Research

Prevention, Primary & Emergent Health Service	General Health Service	Child-Focused Health Service	Children's Designated Health Services	Children's Enhanced & Regional Subspecialty Health Services	Children's Provincial Subspecialty Health Services
T1	T2	T3	T4	T5	T6
Mechanisms in place to regularly review the quality of care, including case reviews. If child involved, physicians & staff with child health expertise are included in the review.<br><br>Participates in regional & provincial child health quality improvement initiatives.	Same as T2.	Same as T2 plus:<br><br>Provides pediatric expertise for T1/T2 case reviews, if requested.	Mechanisms in place to regularly review the quality of care provided to children, including case reviews.<br><br>Identifies relevant regional child health quality indicators. If T5 exists within the HA, works in conjunction with T5.<br><br>Leads/participates in regional/provincial child health quality improvement initiatives.	Same as T4 plus:<br><br>Identifies regional child health quality indicators.<br><br>Leads/participates in regional & provincial child health quality improvement initiatives.<br><br>Participates in research related to pediatric care.	Mechanisms in place to regularly review the quality of care provided to children, including case reviews.<br><br>In collaboration with CHBC & HAs, develops & disseminates guidelines on relevant child health topics.<br><br>Identifies provincial child health quality indicators.<br><br>Leads provincial quality improvement initiatives.<br><br>Conducts & supports others to conduct child health-related research.
